# Case Report: A Recovered SARS CoV-2 Patient Protected From Reinfection

**DOI:** 10.3389/fmed.2020.564264

**Published:** 2020-09-25

**Authors:** Waleed Mahallawi

**Affiliations:** Medical Laboratory Technology Department, College of Applied Medical Sciences, Taibah University, Madinah, Saudi Arabia

**Keywords:** SARS CoV-2, reinfection, protection, herd immunity, vaccines

## Abstract

The COVID-19 pandemic caused by SARS CoV-2 is a worldwide emergency, and is taking a substantial toll on human health, lives, and the global economy. Due to the novelty of this virus, no SARS CoV-2-specific treatments or licensed vaccines are available though few vaccines are undergoing clinical trials. Therefore, continued research into an effective vaccine is an urgent necessity. The reinfection of recovered patients is one of the major concerns of healthcare providers worldwide. Health authorities are currently seeking evidence of protection from reinfection in recovered individuals. This is the first case report in Saudi Arabia on a patient who was diagnosed as COVID-19-positive; recovered; and after successful recovery was protected against reinfection.

## Introduction

The coronavirus disease 2019 (COVID-19) caused by Severe Acute Respiratory Syndrome Coronavirus-2 (SARS CoV-2, formerly known as 2019-nCoV), was first identified in China and has attracted international attention (1). On 30th January 2020, the World Health Organization (WHO) declared the COVID-19 epidemic “a public health emergency of international concern.” On March 11, 2020, the WHO declared the novel coronavirus outbreak “a global pandemic” (2). The appearance of SARS CoV-2, after SARS CoV in 2002 and Middle East Respiratory Syndrome (MERS) CoV in 2012, is notable as the third epidemic emergency of a highly pathogenic human coronavirus in the twenty first century (3). Globally, 19,462,112 confirmed cases of COVID-19, causing 722,285 deaths have been reported as on 09-08-2020 (https://covid19.who.int/). In Saudi Arabia, the total confirmed COVID-19 cases was 288,690 with 252,039 recovered cases and 3,167 deaths, as accessed on 09-08-2020 (https://covid19.moh.gov.sa).

The COVID-19 pandemic remains a serious global challenge. Research into diagnostic tests for SARS CoV-2 continues to progress (4); however, care must be taken in evaluating the tests and interpreting their outcomes (5). The main diagnostic tool is real-time PCR (RT-PCR) with samples such as nasal swabs, tracheal aspirate or bronchoalveolar lavage. Computed tomographic images help in diagnosis as well as follow-up (6).

In the absence of any effective therapeutic intervention for COVID-19, antiviral drugs, chloroquine/hydroxychloroquine and respiratory therapy are being tried. The only intervention apparently effective in lowering the contagion rate of this highly infective and transmissible virus is by avoiding contact and self-isolation/quarantine (7).

The main cause of death from COVID-19 appears to be a cytokine storm that correlates with disease severity (8). Antibody response to COVID-19, particularly IgG and neutralizing antibodies mediate protection by blocking viral entry into the host cells as well as post-viral infection (9). This forms the basis of administration of convalescent sera from recovered patients as a viable therapeutic option to provide immediate immunity to infected patients (10). The role of T cells in immunological memory following recovery from COVID-19 has also been suggested (11).

A vaccine is urgently desired to prevent COVID-19 and thus stall the complications and deaths occurring from community transmission of the infection (12). Several COVID-19 vaccines are under clinical trials (https://www.who.int/docs/default-source/coronaviruse/novel-coronavirus-landscape-covid-19cf1952c105464714aaaf8c7cd5c5cc8b.pdf?sfvrsn=d6073093_5&download=true). It is very important to ensure the immunogenicity, and safety of any vaccine before it is licensed. The most recent vaccine candidate produced by Oxford University called ChAdOx1 nCoV-19 is now in the last phase of clinical trials and has shown optimistic results (13).

While a plethora of studies suggest protection after recovery from COVID-19, there are no reports on protection of recovered humans against re-infection (14, 15). This report aims to document the apparent protection of a recovered COVID-19 patient from re-infection. This is of global interest to the health authorities, who are seeking evidence for protection of recovered individuals against reinfection, in the hope of decreasing the current enormous COVID-19 burden worldwide.

## Methodology

### Subjects

Six members of a family, including a house maid, with no underlying co-morbid diseases, were enrolled in this study. Their prior consent was obtained to report this case study. The study was reviewed by the Ethical Committee of Taibah University, and carried out in accordance with the guidelines of Institutional Review Board (SREC/AMS 2020/12/CLD).

### RT-PCR for SARS CoV-2

For the qualitative detection of SARS-CoV-2, nasopharyngeal swab samples from suspected patients were subjected to the cobas® 6,800 SARS-CoV-2 RT-PCR test using the fully automated cobas® 6,800 (Roche). The test was performed as per the manufacturer's instructions (https://diagnostics.roche.com/global/en/products/params/cobas-sars-cov-2-test.html). Selective amplification of target nucleic acid from the sample was performed using target-specific primers (forward and reverse) for amplifying two regions; ORF1 and the structural protein envelope E-gene of SARS-CoV-2. The reported patient cycle threshold (Ct) value was 22.3 which was considered as positive with high viral load.

### Rapid Test for Detection of COVID-19 IgG

COVID-19 IgG rapid test (LIONEX Diagnostics and Therapeutics, Germany) was performed for the qualitative detection of IgG antibodies to SARS-CoV-2 in serum according to the manufacturer's instructions (https://lionex.de/wp-content/uploads/2020/06/LD_COVID-19_IgG_EN_Instructions-for-use_Rev.-1.0-.pdf. Control samples included serum from patients with flu-like symptoms but negative for SARS CoV-2 RT-PCR.

### Case Description

**Figure 1:**
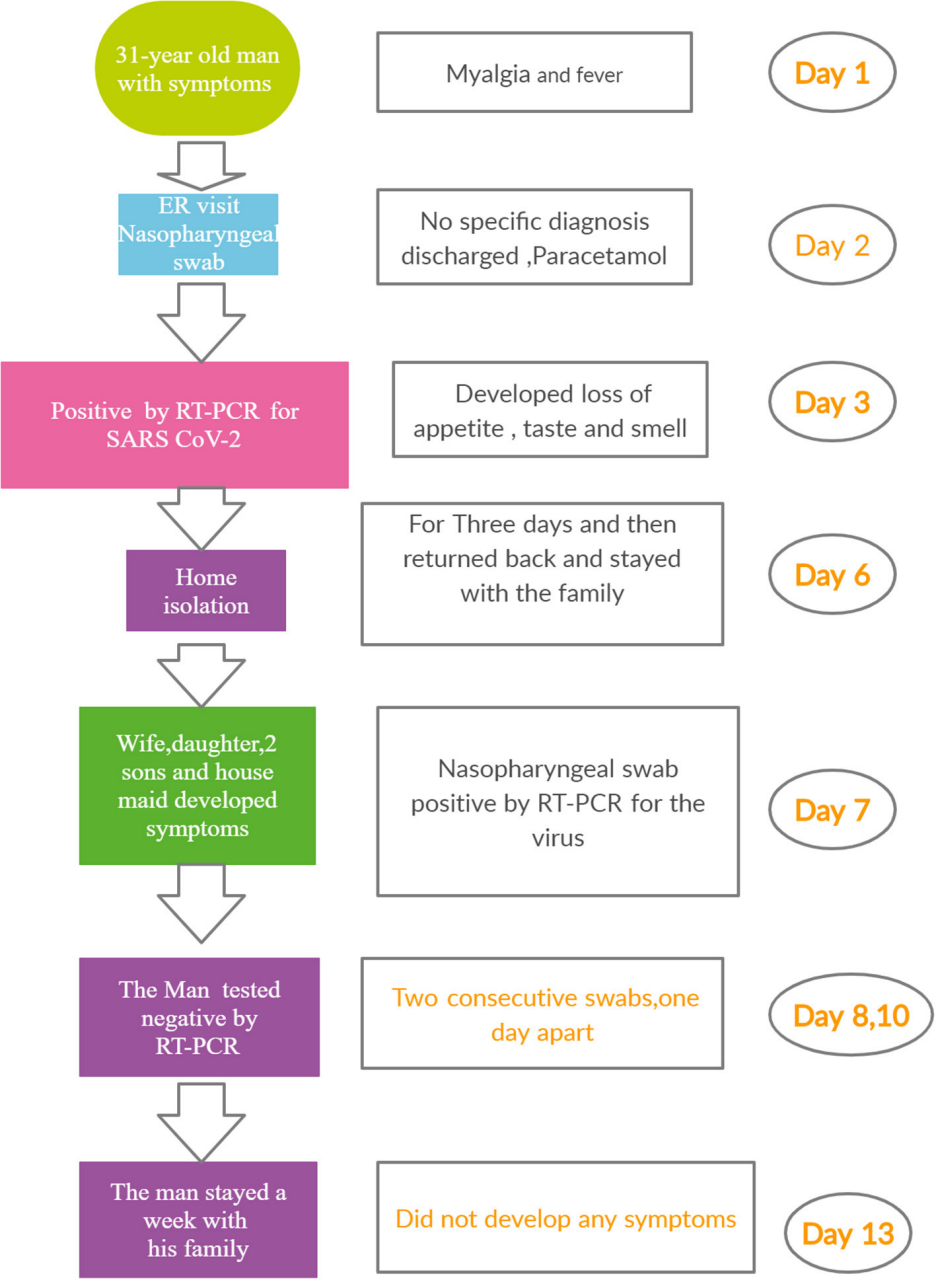
Timeline of case report.

A 31-year-old man presented himself at the Emergency room (ER) of a hospital with myalgia and headache. He had no other symptoms and was not suffering from any chronic disease. His temperature as recorded was 39°C. A nasopharyngeal swab was taken before he was given paracetamol and sent home. The next day, he called the Ministry of Health (MOH) helpline number (937) to seek advice, as he was feeling unwell. He was advised to isolate himself until the laboratory results were available. Subsequently, the MOH staff called him to inform that his RT-PCR test for SARS CoV-2 was positive. Within hours, the MOH staff reached his house. They advised him to stay isolated, As his home was suitable for quarantine/self-isolation, they advised him to stay isolated in a separate room with an attached washroom. The patient did not develop any symptom. Three days after the onset of first symptoms, the patient felt better and stopped taking paracetamol. Unfortunately, he broke isolation and returned to his normal life, living and interacting with his family. Five days later, everyone else at the home (his wife, one daughter, two sons, and a housemaid) developed unusual symptoms. The clinical characteristics of the patients are summarized in Table 1. The man called the MOH staff, who attended and took nasopharyngeal swabs of all five household members. MOH contacted the family and informed them that they were all positive for the virus. The man called the MOH, stating that he felt very well, and requested for a nasopharyngeal swab to be taken. MOH staff attended and took his swab. The next day, they called to inform him that he was negative for CoV-2. The following day, the MOH staff took another swab. Again, the result was negative. The man stayed at home with his family without isolation for another week. At the end of the week, swabs were again taken from all the members of the household, including the original patient. All the results were negative (Figure 1).

**Table 1:** Summary of the symptoms in family members during the course of infection.

**Patient**	**Age (years)**	**Symptoms**	**Symptom interval (days)**
Father	31	Fever (39°C), muscle pain, headache, loss of appetite, taste, and smell	3
Mother	29	Muscle pain, loss of taste, and smell	4
Daughter	10	Fever (38.5°C), loss of appetite	2
Son 1	8	Loss of appetite, vomiting (once)	1
Son 2	2	Loss of appetite	2
House maid	37	Loss of appetite, headache	2

In follow-up investigations on this cohort of six patients, a rapid test was used to screen anti-SARS CoV-2 IgG antibody response. All the six patients showed a consistent pattern of positivity for IgG antibodies one-month post recovery.

## Discussion

Mitigation and defeat of emerging infections have surfaced as the two major public-health approaches in controlling the virus (16). It is vital to reduce COVID-19 infection via minimizing human-to-human contact, which may, however, be publicly and economically unsustainable in the long term (17). A crucial infection control or preventive measure would include development of a vaccine. As of now, no licensed vaccine is available against this novel coronavirus, though quite a few are in various phases of clinical trial (8, 9).

Protection against reinfection post successful recovery from COVID-19 infection would imply the feasibility of development of a vaccine against this virus. Few reports suggest protection after recovery from infection (15, 17). However, in none of the studies, was protection against re-infection explored. This report documents the first case in humans of a recovered patient's protection from reinfection.

The result of this case study is significant in that it suggests that natural infection leads to induction of protection against the virus, at the humoral level. The development of antibodies after infection with COVID-19 is now well-established, however, the appearance of antibodies does not always correlate with a rapid decline in viral load. Normally, in 50% of young to middle-aged COVID-19 patients without underlying major disease, seroconversion occurs 7 days subsequent to infection (18). If this is the case, then herd immunity could be an effective solution for community protection from COVID-19, thus reducing its global impact. It would, therefore, be interesting to test for memory humoral immunity in recovered patients. This would also support the idea that recovered patients' immune response has the capacity to develop persistent antibody production that may help to counter the virus. It also suggests a correlation of protection (from reinfection) with antibody response, and possibly with other cellular components. The vital role of cellular immune response to the virus has been proven in a recent study, wherein, it was found that the extent and degree of memory T cell response from convalescent patients with COVID-19 appeared to be higher in severe compared to mild cases (19).

Additionally, it showed that the overall T cell response as well as the spike protein-specific T cell response correlated with several viral proteins such as spike, receptor binding domain (RBD) and nucleoprotein (NP)-specific antibody end point titer (20).

Moreover, a recent study conducted by the author and co-workers found a massive CD4 T cell response in recovered COVID-19 patients who donated plasma for treatment of severely hospitalized patients (unpublished data).

Till the time of writing of this case report, none of the family members had any unusual symptoms, which suggests that they were all fully recovered. The six patients were also positive for anti-SARS CoV-2 IgG antibodies. This result is consistent with a study which demonstrated anti-SARS CoV-2 IgG antibody in serum of convalescent patient that could neutralize the virus activity in a pseudotype entry assay (21). Additionally, a recent study showed that SARS-CoV-2 infection induced protective immunity against re-exposure in rhesus macaques (22).

The major limitation of this study includes the small sample size. Secondly, a positive PCR result merely implies the recognition of viral RNA, and does not necessarily specify the existence of viable virus (18). Nonetheless, the present case study shows that the immunity of patients who have recovered from infection could permit them to safely interact with vulnerable people in high-contact positions such as healthcare workers. Moreover, verified protection from reinfection would certainly aid the government in reducing the economic pressure and health burden due to the pandemic.

## Conclusion

This case report is the first in humans to show that a recovered patient who had been infected with the novel SARS CoV-2 was protected from reinfection. However, further studies are warranted on a large cohort of COVID-19 recovered patients. This finding may add to the existing concept of protection with convalescent plasma therapy and encourage the scientific community in its renewed efforts in understanding the immune system's interaction with the virus towards development of a successful vaccine.

## Data Availability Statement

The raw data supporting the conclusions of this article will be made available by the authors, without undue reservation.

## Ethics Statement

The studies involving human participants were reviewed and approved by the Ministry of Health, Madinah, Saudi Arabia. The patients/participants provided their written informed consent to participate in this study. Written informed consent was obtained from the individual(s) for the publication of any potentially identifiable images or data included in this article.

## Author Contributions

The author confirms being the sole contributor of this work and has approved it for publication.

## Conflict of Interest

The author declares that the research was conducted in the absence of any commercial or financial relationships that could be construed as a potential conflict of interest.
